# Analysis of worldwide surgical outcomes in COVID-19-infected patients: a gynecological oncology perspective

**DOI:** 10.2144/fsoa-2020-0099

**Published:** 2020-08-21

**Authors:** David L Phelps, Srdjan Saso, Sadaf Ghaem-Maghami

**Affiliations:** 1 Subspecialty Fellow, Gynaecological Oncology Surgery, Hammersmith and Queen Charlotte’s & Chelsea Hospital’s, Du Cane Road, London W12 0HS, United Kingdom; 2 Honorary Clinical Lecturer, Department of Surgery & Cancer, Imperial College London, IRDB, Du Cane Road, London W12 0NN, United Kingdom; 3 Professor of Gynaecological Oncology Surgery, Department of Surgery & Cancer, Imperial College London, IRDB, Du Cane Road, London W12 0NN, United Kingdom; 4 Honorary Consultant Gynaecology Oncology Surgeon, Hammersmith and Queen Charlotte’s & Chelsea Hospital’s, Du Cane Road, London W12 0HS, United Kingdom

**Keywords:** cancer, COVID, COVID-19, gynecological oncology surgery, review, SARS-CoV-2

## Abstract

Coronavirus Disease 2019 (COVID-19) guidance limits all but the most urgent surgery in the United Kingdom. We review the literature and our experience in gynecology to assess perioperative outcomes. PubMed was searched with (surg*[Title])AND(COVID[Title]), (surg*[Title])AND(2019-nCoV[Title]), and (surg*[Title])AND(SARS-CoV-2[Title]), and 67 COVID-19-positive surgical patients across ten hospitals in four countries are included. Median mortality was 33%. Cardiac and pulmonary co-morbidities associated with higher risk of COVID-19-positive postoperative death. Mortality was high in neurosurgery (80%) and the lowest in gynecological oncology surgery (none). This analysis provides an evidence base on which to consider surgical risk assessment for different specialties. Risk of perioperative death needs to be assessed in the context of patients’ co-morbidities and surgical specialty. An individualized approach toward surgical decision making is imperative.

## Lay abstract

Coronavirus Disease 2019 (COVID-19) has resulted in cancer patients receiving inferior surgical treatment compared with normal treatment plans. The risk of death after surgery, when infected with COVID-19, can be as high as 33%. For this reason, during the pandemic, guidelines were issued to protect healthcare services by prohibiting all but the most urgent surgery. This article shows that death rates vary by surgical specialty. The risk of dying within 30 days of surgery is particularly low in gynecological oncology patients. Guidelines should be tailored toward individuals’ needs rather than applying a blanket approach to all.

A novel coronavirus, initially named 2019-nCoV and later renamed severe acute respiratory syndrome coronavirus 2 (SARS-CoV-2), causes Coronavirus Disease 2019 (COVID-19). The first case of COVID-19 was in Wuhan, Hubei province, China, on 17 November 2019. Over the last 5 months, SARS-CoV-2 has swept the world infecting over 7.8 million individuals in 188 countries and is associated with almost 430,000 deaths worldwide [1]. While Asia was the initial epicenter for infections, the virus has since spread globally and deaths associated with the virus are now the highest in the USA, Italy, United Kingdom (UK), Spain and France, respectively [1]. The spectrum of COVID-19 symptoms is wide, with up to 78% being asymptomatic [2]. COVID-19-associated mortality is thought to be around 5.6% [3], but this number has been difficult to ascertain when so many infections can be asymptomatic and thus undiagnozed. This may falsely elevate the denominator and thus the mortality rate.

In the UK, guidelines have been produced and implemented in response to NHS England (Worcestershire, UK) advice to restrict surgical practice during the pandemic for 3 months [4]. These guidelines are intended to reduce pressure on healthcare systems, intensive care units (ICUs), ventilators and to reduce the risk of nosocomial COVID-19 infection and the postoperative sequelae that may ensue. However, while much has been published to guide practice during this pandemic, no review of surgical outcomes in patients with COVID-19 infection had been performed. Since submission of this article, an international collaboration between surgeons and anesthetists (COVIDSurg-Cancer) representing over 800 hospitals in 98 countries has been published [5]. The data presented by the COVIDSurg Collaborative are the most comprehensive analyses of surgical outcomes in COVID-19-positive patients published to date and include over 1000 patients. Surgical cancer cases had a 30-day mortality odds ratio of 1.55 (1.01–2.39, p = 0.046), but the surgical specialties were not analyzed or reported separately [5]. In fact, only 21 cases of the 1128 studied were gynecological with no separate data for gynecological oncology. It is therefore difficult to draw conclusions from the COVIDSurg data regarding gynecological oncology surgery specifically.

It has been suggested that cancer patients are more likely to contract COVID-19 than non-cancer patients. They are more vulnerable, have a higher mortality rate and perhaps should have chemotherapy or surgery postponed [6,7]. However, robust data on the ramifications of perioperative COVID-19 infection in cancer patients, and specifically gynecological oncology patients are lacking.

The restrictions placed on surgery by health departments seem common sense, but there is an ever-growing group of patients in need of surgery with little hope of an operation in the near future. This is concerning for all, but particularly for cancer patients with some hope of curative or life-prolonging surgery. Cancer patients are therefore inevitably undergoing treatment protocols far removed from long-established norms and models of best practice. There is evidence to suggest that these patients are at a significant disadvantage in terms of life-years gained (LYGs) when factoring in a 6-month delay in treatment (18.1 vs 15.9 LYGs) [8]. It has been suggested that urgent cancer diagnostics and surgical pathways should be maintained at current levels to minimize avoidable cancer deaths [8]. One example from our own specialty of surgical gynecological oncology concerns patients with low-grade superficially invasive endometrial cancers, which have cure rates of over 95% after surgery [9]. These tumors are deemed lower surgical priority, in view of often slow rates of progression, despite such high surgical cure rates. Further to this immediate impact on treatment will be the long-term effects caused by further surgical delay when working through the back-log of operations post-COVID-19.

This article reviews all published surgical outcomes with known COVID-19 infection prior to the COVIDSurg publication but particularly focuses on gynecological oncology outcomes. We describe our own experience and outcomes when operating on two patients with COVID-19 in our gynecological cancer center.

## Methods & case histories

We aimed to identify all relevant research articles related to surgical outcomes during the COVID-19 pandemic. We searched Medline (via PubMed) using the search strings: (surg*[Title]) OR (COVID[Title]), and (surg*[Title]) OR (2019-nCoV[Title]), and (surg*[Title]) OR (SARS-CoV-2[Title]). In total, 192 articles were suggested. All the authors independently screened the articles by title and abstract. Of all articles, 14 were read in full and six were included for the purposes of this review [10–14].

Details of our own cases were collected retrospectively from electronic hospital systems and are presented here and summarized in Table 1.

**Table 1.** Summary of the two patients who underwent surgery to treat a gynecological cancer at our center.

Variables	Patient 1	Patient 2
**Age range (years)**	50–60	50–60
**Diagnosis**	Squamous cell carcinoma of vulva	History of choriocarcinoma with cervical lesion
**Surgery**	Supraradical vulvectomy, distal urethrectomy and vaginectomy with anterolateral thigh flap reconstruction	Laparotomy, modified radical hysterectomy and bilateral salpingo-oophorectomy
**Co-morbidities**	Obesity, hypertension, Type 2 diabetes mellitus, asthma, cancer	Previous choriocarcinoma
**Timing of diagnosis after surgery (days)**	19 (and 16 days post-COVID-19)	6
**Symptoms**	Fever	Cough, fever
**Heart rate (highest)**	109	124
**O_2_ sats (lowest)**	94%	95%
**White cell count at COVID-19 diagnosis**	7.0 × 10^9^	2.3 × 10^9^
**Neutrophils at COVID-19 diagnosis**	5.3 × 10^9^	0.9 × 10^9^
**C-reactive protein at COVID-19 diagnosis**	175 mg/l	120 mg/l
**Chest XR**	Lower left lobe ground glass opacification	Normal
**ICU admission**	Yes	No
**Outcome**	Recovered from COVID-19 and remains well	Recovered from COVID-19 and remains well

COVID-19: Coronavirus Disease 2019; ICU: Intensive care unit; XR: XRay.

Patient-1 presented with advanced squamous cell carcinoma of the vulva. She underwent a supraradical vulvectomy, distal urethrectomy and distal vaginectomy with anterolateral thigh flap reconstruction and partial thickness skin grafting to the thigh. Further refashioning surgery was planned 3 weeks later. Initial recovery was as expected but on day 19 the patient developed a fever of 37.9°C and tested positive for COVID-19 with reverse transcriptase PCR (RT-PCR). Oxygen saturations were 94% at the time of COVID-19 diagnosis, with a heart rate of 109 beats per minute. The patient was otherwise asymptomatic. White cell count was 7.0 × 10^9^ (normal range: 4.0–11.0 × 10^9^) and falling and neutrophils were 5.3 × 10^9^ (normal range 2.0–7.5 × 10^9^), despite a significantly raised C-reactive protein of 175 mg/l (normal range: <10 mg/l). Chest imaging was normal. The patient’s co-morbidities included obesity, hypertension, Type 2 diabetes mellitus and asthma. She was treated on the ward and isolated appropriately. Refashioning surgery was delayed due to COVID-19 infection. On day 35, the patient returned to theater for vulval refashioning after a negative COVID-19 swab and went to the ICU for recovery as a precaution for 24 h. Further refashioning surgery was performed 18 days later. Her recovery postoperatively was unremarkable from a COVID-19 perspective.

Patient-2 presented with a history of choriocarcinoma with an abnormal mass in the cervix and underwent an open radical hysterectomy. On day 3, the patient developed a cough and fever (37.9C) with oxygen saturations of 95% and a heart rate of 124. She tested positive for COVID-19 by RT-PCR on day 6. White cell count had reduced to 2.3 × 10^9^ and neutrophil count was 0.9 × 10^9^, despite C-reactive protein of 120 mg/l. Chest imaging was normal. She was otherwise well and was discharged home on day 9. The patient had no significant co-morbidities aside from choriocarcinoma.

## Results

The data collected and analyzed from all studies are summarized in Tables 2 and 3. 65 surgical patients with perioperative COVID-19 infection were identified in the literature and included in this review. Additionally, two cases at our own hospital were included, totaling 67 patients. Patients were treated at ten hospitals in four countries (China, Italy, Iran and UK) and ranged from 21 to 84 years of age. Females comprised 52.2% of the patients. Of the 67 patients undergoing surgery, 20 died (median mortality: 33%). Fever was noted in 95.2% (59/62), dyspnea in 58.1% (36/62) and cough in 56.5% (35/62) of COVID-19-positive surgical patients from studies that reported symptoms, including our own data. The largest study in this series reported no difference in the pattern of symptoms between severe and non-severe (ICU vs non-ICU) patients [12].

**Table 2.** Study summary.

Study (year, location)	Patient number	Study duration	Median age/female	Operative details	ICU care/mortality	General findings
**Aminian *et al.* (2020)** (Tehran, Iran) SC, R	Four diagnosed but one excluded Study number = 3 (one hospital)	02/2020	75 (54–81)/n = 2 (66.6%)	Cholecystectomy: n = 1 (25%) Hernia repair: n = 1 (25%) Gastric bypass: n = 1 (25%) Hysterectomy: n = 1 (25%)	NS; n = 1 (NS/33.3%)	COVID-19 can complicate the perioperative course with diagnostic challenge and high potential fatality rate In locations with widespread infections and limited resources, risk of elective surgical procedures for index patient and community may outweigh the benefit In some situations, postponing elective surgical procedures might be right decision Another option would be routine or selective screening of patients for COVID-19 before elective surgical procedures
**Cai *et al.* (2020)** (Wuhan, China) SC, R	Study number = 7 (one hospital)	01/01/2020 – 22/01/2020	60 (57–68)/n = 5 (71.4%)	Surgical time (median, range): 165 min (110–280) Blood loss (median range): 100 ml (50–200) VATS lobectomy: n = 4 (57.1%) VATS segmentectomy plus wedge resection: n = 1 (14.2%) Thoracotomy sleeve lobectomy: n = 1 (14.2%) Lobectomy plus bronchus reconstruction: n = 1 (14.2%)	n = 3; n = 3 (42.9%/42.9%)	Outbreak imposes major challenge in deciding and managing surgical operation on patients with lung cancer and other lung disorders Preliminary results from limited cases indicate that lung resection surgery might be a risk factor for death in patients with COVID-19 in the perioperative period
**Lei *et al.* (2020)** (Wuhan, China) MC, R	37 diagnosed but three excluded Study number = 34 (four hospitals)	01/01/2020 – 05/02/2020	55 (21–84)/n = 20 (58.8%)	Surgical time (median; IQR): 178 (70–249) min ICU vs non-ICU: 200 vs 70 Breast: n = 2 (5.9%) Caesarean: n = 5 (14.7%) GI: n = 12 (35.3%) Gyne: n = 1 (2.9%) Neuro: n = 3 (8.8%) Ophthalmic: n = 1 (2.9%) Ortho: n = 6 (17.6%) Pulmonary: n = 3 (8.8%) Renal transplant: n = 1 (2.9%) Endoscopic: n = 10 (29.4%) Moderate-/high-risk: n = 22 (64.7%) (ICU vs non-ICU: 93.4 vs 42.1%)	n = 15; n = 7 (44.1%/20.5%)	Compared with non-ICU patients: ICU patients older, more likely to have underlying co-morbidities, underwent more complex surgeries and had more severe lab abnormalities (hyperleukocythemia, lymphopenia)
**Li *et al.* (2020)** (Wuhan, China) SC, R	Study number = 13 (one hospital)	01/01/2020 – 20/02/2020	60.2 ± 5.6/n = 3 (23.1%)	Thoracic operations Lung: n = 11 Oesophagus: n = 2	n = 7; n = 5 (46.2%; 38.5%)	COPD significantly associated with severity and death Chest operation was significantly associated with death COVID-19 is associated with poor prognosis for patients undergoing thoracic operation, especially for those with COPD Implementation of comprehensive protective measures is important to control nosocomial infection
**Panciani *et al.* (2020)** (Brescia, Italy) SC, R	Study number = 5 (one hospital)	21/02/2020 – 23/03/2020	82 (77–86)/n = 0 (0%)	Neurosurgery: n = 3 (Craniotomy: n = 2) (Burr hole: n = 1) Endovascular treatment: n = 2 All for chronic subdural hematoma	NS; n = 4 (NS; 80%)	The authors observed mortality rate of 80% about 21.6-times greater than control data
**Yang *et al.* (2020)** (Wuhan, China) SC, R	Study number = 3 (one hospital)	01/01/2020 – 20/02/2020	50.1 (mean)/n = 3 (100%)	Hysterectomy: n = 1 Radical hysterectomy: n = 1 Cytoreductive surgery: n = 1	n = 0; n = 0	Severe infection may be related to older age, co-morbidities, malignant tumor and surgery in gynecologic hospitalizations Given the long and uncertain incubation period of COVID-19, screening for the virus infection should be carried out for all patients, both preoperatively and postoperatively Postponement of scheduled gynecologic surgery for patients in the epidemic area should be considered

COPD: Chronic obstructive pulmonary disease; COVID-19: Coronavirus Disease 2019; ICU: Intensive care unit; IQR: Interquartile range; GI: Gastrointestinal; MC: Multicenter; NS: Nonsignificant; R: Retrospective; SC: Single center; VATS: Video assisted thoracic surgery.

**Table 3.** Patient characteristics.

Study (year)	Co-morbidities (ICU vs non-ICU)	SAR-CoV-2 diagnosis	Symptoms	Complications (ICU vs non-ICU)	Treatments (ICU vs non-ICU)
**Aminian *et al.* (2020)**	Obesity: n = 1 (33.3%)	Lab confirmation done by quantitative RT-PCR on samples from nasopharynx	Abdo pain: (n = 1; 33.3%) Diarrhea: (n = 1; 33.3%) Dry cough: (n = 1; 33.3%) Dyspnea: (n = 3; 100%) Fever: (n = 3; 100%) Vomiting: (n = 1; 33.3%)	ARDS: n = 2 (66.6%) ACI: n = 1 (33.3%)	NS
**Cai *et al.*** **(2020)**	Smoking: (n = 4; 57.1%) COPD: (n = 2; 28.6%) CVD: (n = 3; 32.4%) ILD: (n = 1; 14.3%)	Lab confirmation done by quantitative RT-PCR on samples from respiratory tract Time of surgery to first symptom: (IQR; median) 7.0 (7.0–10.0) days	Diarrhea: (n = 1; 14.3%) Dry cough: (n = 4; 57.1%) Dyspnea: (n = 5; 71.4%) Fever: (n = 7; 100%) Fatigue: (n = 2; 28.6%) Myalgia: (n = 2; 28.6%) Palpitation: (n = 1; 14.3%)	NS	Antivirals/antibiotics n = 7 (100%) Corticosteroids n = 4 (57.1%) IMV n = 3 (32.4%) Immunoglobulins n = 3 (42.9%) Oxygen n = 7 (100%)
**Lei *et al.*** **(2020)**	Any: n = 20 (58.8%) (80 vs 42.1%; p = 0.04) HTN: n = 13 (38.2%) (60 vs 21.1%; p = 0.03) CVD: n = 7 (20.6%) (40 vs 5.3%; p = 0.03) Other (p > 0.05, NS): Malignancy: n = 9 (26.5%) Diabetes: n = 8 (23.5%) CBD: n = 2 (5.9%) COPD: n = 1 (2.9%) CKD: n = 1 (2.9%) Moderate-/high-risk surgery: n = 22 (64.7%) (93.4 vs 42.1%; p < 0.05)	Lab confirmation done by quantitative RT-PCR on samples from respiratory tract Time of surgery to first symptom: (IQR; median) 2.0 (1.0–4.0) days First symptom to dyspnea: (IQR; median) 3.5 (2.0–5.3) days	Five most common: Dry cough: (n = 18; 53%) Dyspnea: (n = 15; 44.1%) Fatigue: (n = 25; 74%) Fever: (n = 31; 91%) Myalgia: (n = 11; 32.4%) No significant difference between ICU and non-ICU patients	ARDS: n = 11 (32.4%) (60 vs 10.5%; p = 0.003) Shock: n = 10 (29.4%) (53.3 vs 10.5%; p = 0.01) 20 infection: n = 10 (29.4%) (46.7 vs 10.5%; p = 0.01) ACI: n = 5 (14.7%) (33.3 vs 0%; p = 0.01) Other (p > 0.05, NS): Arrhythmia: n = 8 (23.5%) AKI: n = 2 (5.9%)	Antivirals/antibiotics n = 34 (100%) Glucocorticoids n = 16 (47.1%) Immunoglobulins n = 14 (41.2%) CKRT n = 1 (2.9%) Oxygen n = 19 (55.9%) NIV n = 10 (29.4%) IMV n = 5 (14.7%) ECMO n = 1 (2.9%)
**Li *et al.*** **(2020)**	Smoking: n = 7 (53.8%) HTN: n = 2 (15.4%) Diabetes: n = 1 (7.7%) COPD: n = 5 (38.5%) CHD: n = 4 (30.8%)	Lab confirmation by quantitative RT-PCR on samples from respiratory tract Days of exposure to onset of fever: 5.9 ± 4.7 days	Cough: n = 10 (76.9%) Diarrhea: n = 2 (15.4%) Dyspnea: n = 12 (92.3%) Fatigue: n = 8 (61.5%) Fever: n = 13 (100.0%)	NS	NS
**Panciani *et al.*** **(2020)**	NS	Lab confirmation by quantitative RT-PCR on samples from nasopharynx	NS	Thrombocytopenia: n = 2 (40%) Interstitial pneumonia: n = 5 (100%)	Antivirals/antibiotics n = 5 (100%) Hydroxychloroquine n = 5 (100%)
**Yang *et al.*** **(2020)**	Diabetes: n = 2 (66.7%) HTN: n = 3 (100%)	Lab confirmation by quantitative RT-PCR on samples from throat Days of exposure to onset of fever: 6 days Time of surgery to first symptom: 1.3 days	Cough: n = 1 (33.3%) Dyspnea: n = 0 (0%) Fever: n = 3 (100.0%)	NS	Antivirals/antibiotics n = 3 (100%)

ACI: Acute cardiac injury; AKI: Acute kidney injury; ARDS: Acute respiratory distress syndrome; CBD: Cerebrovascular disease; CHD: Coronary heart disease; CKD: Chronic kidney disease; CKRT: Continuous kidney replacement therapy; COPD: Chronic obstructive pulmonary disease; CVD: Cardiovascular disease; ECMO: Extracorporeal membrane oxygenation; HTN: Hypertension; ICU: Intensive care unit; ILD: Interstitial lung disease; IMV: Invasive mechanical ventilation; IQR: Interquartile range; NIV: Noninvasive ventilation; NS: Nonsignificant; RT-PCR: Reverse transcriptase PCR.

From four studies – plus our own data – which reported ICU admissions of surgical patients (59 patients), 26 were admitted to ICUs (44.1%) and of those patients 15 died (15/26; 57.7% of ICU admissions). Any co-morbidity appears to increase the risk of ICU admission (p = 0.04) [12]. Particularly co-morbidities affecting the heart (hypertension, coronary heart disease, cardiovascular disease) and the lungs (smoking, interstitial lung disease and chronic obstructive pulmonary disease) appeared to influence ICU admission the most [10–13]. Age was an important risk factor for contracting COVID-19 infection postoperatively with the median age in all studies greater than 50.

Thoracic surgery, particularly lung resection and in patients with chronic obstructive pulmonary disease, is associated with high morbidity and mortality (38–42.9%) [11,13]. Furthermore, mortality after neurosurgery was 80%, 21.6-times greater than in COVID-19-negative neurosurgical control patients [15], although this study only included four patients. Three studies included gynecological patients [10,12,14] and when added to our own cases, totaled seven gynecological patients, six of whom had confirmed cancer. Operations were major (ovarian cytoreduction, n = 2; radical hysterectomy, n = 2; hysterectomy, n = 2; supraradical vulvectomy, n = 1). A further five women had cesarean sections [12]. None of these 12 major obstetric and gynecological surgery patients with COVID-19 died post-surgery [10,12,14].

## Discussion

As we plan for recovery from the COVID-19 pandemic, we need to define morbidity and mortality from surgery for urgent and time-sensitive conditions, including cancer. This will help inform surgeons and patients in decision making. All the studies published thus far during the pandemic have shown a substantial increase in mortality from COVID-19 infection in the perioperative period compared with patients who did not have surgery.

COVIDSurg data report a perioperative mortality rate of 23.8% in COVID-19-infected patients. Risk factors for mortality were the highest in male gender, age >70, multiple co-morbidities, malignant diagnosis and major emergency surgery [5]. The currently published data are not granular enough to comment on the exact reasons for observations but particularly have few gynecological oncology cases to draw firm conclusions. Cancer and most other medical conditions requiring urgent surgery are linked with increased COVID-19-related deaths in any case. Major surgery is likely to result in some degree of immune suppression, making patients more prone to infection and its complications.

The studies presented here do not necessarily distinguish between patients who may have had an active COVID-19 infection when they were operated on from patients who were colonized, or simply acquired the infection immediately postoperatively, at the hospital. It is possible that a colonized airway at the time of intubation may lead to significant dissemination of the virus in the lungs resulting in much more severe pulmonary complications. Measures have been introduced to reduce the risk of hospital acquisition of the virus and to identify which patients may have colonization or asymptomatic infection. Examples include introduction of COVID-19-free operating sites, testing patients for the SARS-CoV-2 RNA 48 h preoperatively and the concept of COVID-19 free ‘teams’ who are tested regularly and do not care for COVID-19-positive patients.

As it is the case for nonoperative COVID-19-related death, from our analysis, perioperative death from COVID-19 is also associated with pre-existing patient co-morbidities. This should be a consideration in preoperative case selection and patient counseling for nonemergency cases. Thoracic and neurosurgical procedures had the highest COVID-19-related mortality rates, although the numbers are too small to draw any clear conclusions from this.

Our review has identified five women who had pericesarean COVID-19 infections and they also reported no mortality. The Royal College of Obstetricians and Gynaecologists has recommended that the obstetric management of elective cesarean birth should be according to usual practice [16]. Gynecological procedures, including radical gynecological oncological surgery, reported no perioperative deaths. However, this observation is based on only seven patients and this observation may be due to the gender-related susceptibility to COVID-19, although in other papers mortality rates were comparable between men and women [12]. Despite limited evidence and small case numbers, the data from gynecological surgery are reassuring, despite most patients having treatment for cancer. The British Gynaecological Cancer Society has been selective in advising and recommending surgery to women with the most time-sensitive cancers [17]. This was appropriate at the peak of the pandemic but as we move past the peak, and clean sites and Cancer Hubs are established, we may wish to consider operating on more cancer patients with less aggressive tumors or on patients that require more extensive surgery.

## Conclusion

Whilst the case numbers are relatively small in this review, we highlight that perioperative mortality, when infected with COVID-19, varies considerably by surgical specialty. In specific situations it may be reasonable to proceed with planned surgery, during the COVID-19 pandemic, in specific situations. Surgery should still be considered especially when long-term survival could be negatively affected by delaying surgery. COVID-19-specific informed consent should be considered for all but the most urgent cases. Individualizing treatments for patients, balancing risks and benefits for each patient for each condition, and considering patients’ age and co-morbidities remain paramount when decision-making for doctors and patients.

## Future perspective

COVID-19 has affected every facet of society, but its effects have perhaps been most significantly felt within the healthcare systems of the world. While some countries have had success in containing the first wave of the virus, there appear to be second, third or worse waves to come. A lot can be learned from the first few months of this pandemic, but we must learn from these difficult times to ensure that healthcare services are not unduly disrupted in any further outbreaks of the virus. Our article suggests that perioperative mortality from COVID-19, even in gynecological cancer patients, is low. This should shape our practice in the future as we learn to live with COVID-19 in the medium- to long-term.

**Executive summary**

**Background**

- Coronavirus Disease 2019 (COVID-19) has had an exceptional impact on healthcare services worldwide, especially surgical oncology services.
- Guidelines have been introduced to limit surgery often to only emergency cases, leaving cancer patients with potentially inferior treatment plans.
- No analysis of perioperative surgical outcomes in gynecological oncology patients has been performed until now.

**Aim**

- This article endeavored to highlight gynecological oncology surgical cases with perioperative COVID-19 infection to assess outcomes.
- Our own experience of perioperative COVID-19 infection in a London gynecological oncology cancer center is included.

**Conclusion**

- Perioperative COVID-19 infection appears to result in up to 33% mortality.
- Mortality associated with COVID-19 seems to be specialty specific, with no perioperative deaths in gynecological oncology cases in the published literature and our own data.
- Blanket-approach guidelines for managing surgical services in the midst of a pandemic should be avoided. Guidelines should aim to provide specialty-specific guidance to ensure that high-risk surgical oncology patients, with low COVID-19-associated mortality, continue to receive optimal surgical care.
